# A DNA Damage Repair Gene Signature Associated With Immunotherapy Response and Clinical Prognosis in Clear Cell Renal Cell Carcinoma

**DOI:** 10.3389/fgene.2022.798846

**Published:** 2022-05-17

**Authors:** Linjie Peng, Jiaming Liang, Qi Wang, Guodong Chen

**Affiliations:** ^1^ Organ Transplant Center, The First Affiliated Hospital, Sun Yat-sen University, Guangzhou, China; ^2^ Guangdong Provincial Key Laboratory of Organ Donation and Transplant Immunology, The First Affiliated Hospital, Sun Yat-sen University, Guangzhou, China; ^3^ Guangdong Provincial International Cooperation Base of Science and Technology (Organ Transplantation), The First Affiliated Hospital, Sun Yat-sen University, Guangzhou, China; ^4^ Department of Internal Medicine, The Second Affiliated Hospital of Guangzhou Medical University, Guangzhou, China; ^5^ State Key Laboratory of Respiratory Disease, National Clinical Research Center for Respiratory Disease, The First Affiliated Hospital of Guangzhou Medical University, Guangzhou, China

**Keywords:** clear cell renal cell carcinoma, DNA damage repair genes, immunotherapy response, prognosis, survival

## Abstract

**Background:** Clear cell renal cell carcinoma (ccRCC) is the most common subtype in renal cell carcinoma with relatively poor clinical outcomes DNA damage repair genes (DDRGs) as potential biomarkers are rarely reported in predicting immunotherapy response and clinical prognosis for ccRCC.

**Methods:** RNA-seq and clinical data of ccRCC cohort were collected form TCGA database. Univariate Cox regression and LASSO analysis were performed to construct a DDRG risk signature. Functional enrichment analysis was performed to explore latently enriched pathways associated with DDRG signature. Immune cell infiltration level was estimated using gene set enrichment analysis, and immune response of ccRCC was predicted by tumor immune dysfunction and exclusion (TIDE) algorithm. To predict 1-, 3-, and 5-years overall survival (OS), a nomogram was constructed based on independent prognostic factors, whose performance would be evaluated by calibration curve.

**Results:** A total of 47 DNA damage repair related genes (DDRGs) with significant prognostic value were identified in the ccRCC cohort (*n* = 519). A DDRG risk signature comprising six DRRGs (MSH3, RAD54L, RAD50, EME1, UNG, and NEIL3) were constructed by the LASSO analysis. ccRCC patients were then divided into low- and high-risk groups based on the risk score. Survival analysis revealed that patients in high-risk groups exhibited significantly poorer OS and progression-free survival (PFS), as was confirmed by the testing dataset. Functional enrichment analysis indicated that differentially expressed genes (DEGs) between high- and low-risk groups were mainly associated with immune-related biological processes in ccRCC, among which the immunodeficiency pathway was significantly enriched in the high-risk group. Though the risk signature was significantly correlated with the immune cell infiltration, PD-1 and PD-L1 were less expressed in the DDRG signature, which might indicate the poor response to immunotherapy in the high-risk group. Furthermore, the Cox regression analysis indicated that the DDRG signature can be served as an independent prognostic predictor when compared to clinical characteristics. Based on the independent prognostic predictors, we constructed a nomogram with excellent predictive ability in OS prediction for ccRCC patients.

**Conclusion:** We developed a reliable DDRG risk signature that can independently predict the OS and PFS of ccRCC, which is also promising for predicting immunotherapeutic responses in ccRCC patients.

## Background

Renal cell carcinoma (RCC) is a common cancer in the urinary system, accounting for >90% of cancers in the kidney and approximately 2% of cancer deaths (Hsieh et al., 2017; Siegel et al., 2019). Clear cell RCC (ccRCC) is the most frequent subtype with a poor prognosis, taking up more than 70% of RCC (Nerich et al., 2014). For localized ccRCC, surgical resection is the first-line treatment, but metastatic recurrence during the follow-up could be found in 30–40% of patients (Ghatalia et al., 2019). Systemic therapies including immunotherapy, targeted therapy, and chemotherapy have been approved for managing advanced ccRCC (Atkins and Tannir, 2018). Given that a percentage of patients respond poorly to systemic therapies, identifying high-risk patients and developing personalized treatment are expected to achieve long-term survival in ccRCC. However, clinical characteristics such as the TNM stage are insufficient to predict ccRCC prognosis and therapeutic response (Warren and Harrison, 2018). Thus, continued efforts are required to explore reliable biomarkers to predict prognosis and immunotherapeutic response for ccRCC.

The role of DNA damage repair genes (DDRGs) in tumorigenesis and progression has been widely investigated (Gourley et al., 2019). DNA damage repair mainly includes mismatch repair, base excision repair, nucleotide excision repair, and homology directed repair, which are indispensable to genetic stability. But the defective DNA damage repair might lead to accumulated genome instability and tumorigenesis (Gavande et al., 2016). The characteristics of DDRGs have been used for cancer treatment. For example, as a well-recognized sensor of DNA damage is the poly ADP-ribose polymerase (PARP), PARP inhibitors have demonstrated exquisite sensitivity to BRCA1/2 mutant cells and tumors (Brown et al., 2017). Additionally, immunotherapy such as PD-1 and PD-L1 have improved survival in a subset of cancer patients, and genomic signature correlates with the response to immune checkpoint therapies (Miao et al., 2018). However, the role of DDRGs in maintaining genome stability and predicting immunotherapeutic response is rarely reported in ccRCC.

Herein, we analyzed the RNAseq data and clinical characteristics downloaded from the TCGA database to comprehensively explore the prognostic role of DDRGs in ccRCC. We tried to identify a prognostic signature comprising DDRGs, and evaluate its role in immunotherapy response. Moreover, to better predict the 1-, 3-, and 5-years overall survival, we constructed a nomogram for ccRCC based on prognostic signature and prognostic clinical characteristics.

## Methods

### Data Acquisition

The RNA-seq data, clinical information, mutation data, and annotation files of the TCGA ccRCC cohort were downloaded from Santa Cruz Xena Browser (https://xenabrowser.net/). After screening, cases with missing clinical data or a survival time of less than 30 days were excluded and a total of 519 ccRCC patients were included in the following analysis. Moreover, the probe IDs of the ccRCC cohort were transformed into gene symbols according to the annotation files.

### Establishment and Validation of a DDRG Risk Signature

For RNA-seq data, we divided the ccRCC cohort into high and low expression groups according to the median expression of DNA damage repair genes (DDRGs). A total of 105 well-studied DNA damage repair genes (DDRGs) from 5 DNA Damage Repair pathways were considered for the establishment of the DDRG model. DDRGs with significant prognosis value in univariate Cox regression analysis (*p* < 0.05) would be further assessed. In order to reduce the over-fitting effect, as well as to enhance the forecast accuracy and explainability of the DDRG model, last absolute shrinkage and selection operator (LASSO) regression was then performed to explore significantly prognostic DDRGs. LASSO-penalized Cox analyses were performed using the R package “glmnet”.

Overall ccRCC dataset (*n* = 519) from TCGA database were randomized 7:3 into training (*n* = 363) and testing dataset (*n* = 156). The training dataset was applied to construct DRRGs model, while the entire dataset and testing dataset were used to validate this established model. The risk score for each patient was calculated as follows: risk score = −0.581× MSH3 + 0.075 × RAD54L − 0.010 × RAD50 + 0.189 × EME1 + 0.197 × UNG +0.104 × NEIL3.

The median risk score was set as cut-off values, and patients were then allocated into high-risk and low-risk groups. Survival analysis between two groups and correlation between the risk scores and clinicopathological features would be assessed.

### Mutation Landscape Analysis

TCGA ccRCC mutation data containing 370 tumor samples were acquired from the R package “TCGAmutations”. The mutation landscape for the six signature DDRGs in ccRCC was visualized using the R package “Maftools” (Mayakonda et al., 2018).

### Functional Enrichment Analysis

The R package “GSVA” was used to estimate the pathway activity for ccRCC patients based on the given genesets.

Then R package “limma” was used to identify differentially expressed genes (DEGs) between low- and high-risk groups (Ritchie et al., 2015), cutoff criteria were defined as |logFC| > 1 and adjusted *p* value <0.05. The R package “clusterPofolier” was used to perform GO and KEGG enrichment analysis for DEGs between the two risk groups (Yu et al., 2012). The significant pathways were determined by a cutoff value of adjusted *p* value <0.05. Furthermore, gene set enrichment analysis (GSEA) was performed to find the significantly enriched pathways in the high- and low-risk groups. A *p* < 0.05 and FDR <0.05 were considered as statistically significant.

### Estimating Immune Cells Infiltration in ccRCC

To explore the correlation between the expression of the signature genes and immune checkpoint markers in CCRCC patients, a set of marker genes defining immune cell types was attained from a previous study (Charoentong et al., 2017). Subsequently, the ssGSEA algorithm from the R package “GSVA” was used to determine the infiltration level of each immune cell type in ccRCC using the gene expression profiles (Hänzelmann et al., 2013). The relationship between the characteristic gene expression and the immune cell infiltration score was also evaluated.

### Predicting Immunotherapy Response

The R package ‘maftools’ was used to evaluate the mutation landscape of the TCGA ccRCC cohort. The TMB was measured according to tumor-specific mutated genes (Budczies et al., 2019). The response of each sample to PD-1/PD-L1 and CTLA4 inhibitors was evaluated according to the gene expression profiles of the ccRCC cohort with the Tumor Immune Dysfunction and Exclusion (TIDE) algorithm (http://tide.dfci.harvard.edu) (Jiang et al., 2018).

### Construction and Evaluation of Nomogram Model

Based on independent prognostic indicators in the Cox regression analysis, a nomogram was constructed with indicators like age, pathological grade, clinical stage, distant metastasis, and DDRG signature score using R package “rms”. We first identified the clinical characteristic with prognostic value by univariate cox regression. Next, we integrated the prognostic clinical characteristic and gene signature to construct the nomogram by multivariate cox regression. To evaluate the predictive ability of the nomogram, calibration curves were constructed to demonstrate the consistency between observed and predicted outcomes of 1-, 3-, and 5-year OS in ccRCC patients. The *x*-axis of the calibration curve represents the predicted survival probability and the *y*-axis represents the actual survival probability. Generally, if the predicted survival probability falls into the ideal survival probability line, we can conclude that the prediction model is of good performance.

### Survival Analysis

The ccRCC samples were classified into either high/low risk groups based on the median risk score. Univariate Cox regression and multivariate stepwise Cox regression were used to evaluate the prognostic factors between low- and high-risk groups. Clinical data like age, gender, pathological grade, clinical stage, distant metastasis, lymph node invasion, and tumor size, as well as DDRG data, would be enrolled in the analysis. Kaplan–Meier curve and log-rank test were used to compare overall survival (OS) and progression-free survival (PFS) between two groups. *p* ≤ 0.05 was defined as significant. All the survival analyses and log-rank tests were performed using the R package “survival”, while the R package “surviminer” was used to plot the Kaplan–Meier curve.

### Statistical Analysis

Pearson’s correlation test was used to assess the relationship between the risk score and immune markers, characteristic gene expression, and the immune cell infiltration score, respectively. Fisher’s exact test or chi-square test was performed for comparison of categorical data, while the student’s t tests were used for continuous data. Statistical significance was defined as *p* < 0.05. All statistical analyses were performed in the R environment (R version: 4.0.2).

## Results

### Identification of Six Prognostic DDRGs and Validation of DDRG Risk Signature

**FIGURE 1:**
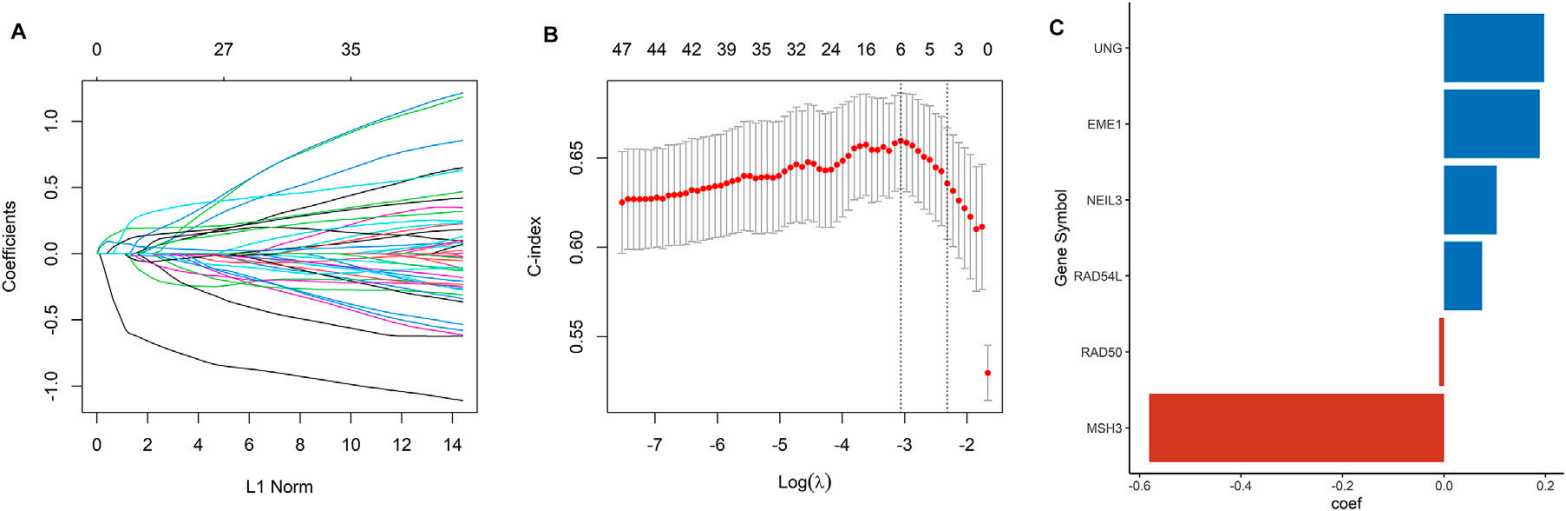
Construction of a DDRG risk signature. **(A)** LASSO coefficient profiles for the 47 DEGs. **(B)** Ten-fold cross-validation of the LASSO analysis. **(C)** The coefficient value of the six DDRGs selected by the LASSO analysis.

A total of 105 DDRGs were investigated, 47 of which were significantly correlated with OS in univariate Cox regression analysis and considered for constructing DDRG signature. LASSO-penalized Cox analysis then effectively discerned the 6 most available prognostic biomarkers to independently predict OS outcomes from the 47 prognostic DDRGs (Figures 1A, B). Coefficients of six DDRGs (MSH3, RAD54L, RAD50, EME1, UNG, and NEIL3) in the training dataset were used to calculate the risk score of ccRCC patients, and the DDRG signature was thus constructed (Figure 1C).

**FIGURE 2:**
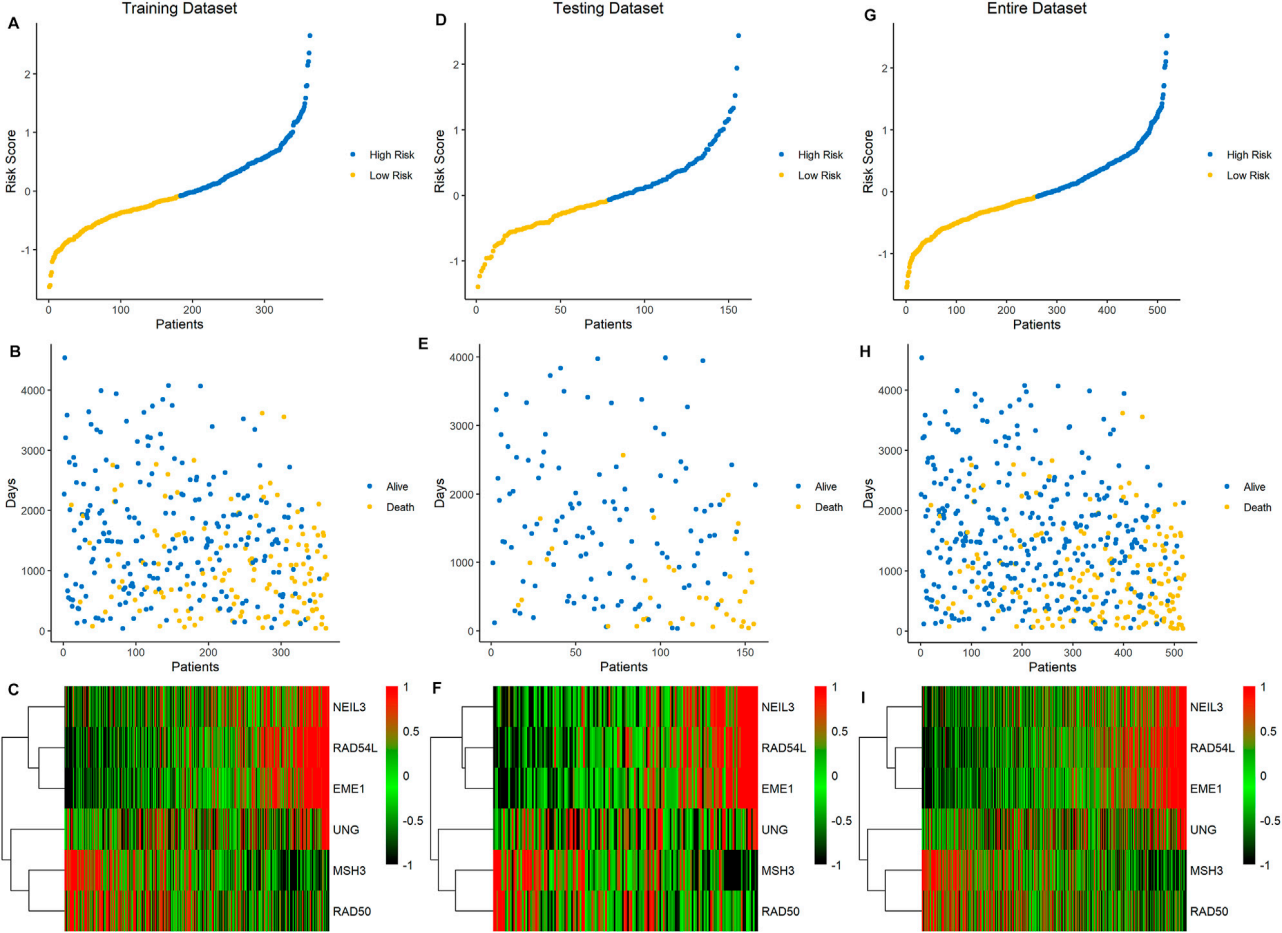
Association between the risk score of six DDRG signatures and ccRCC patients in three cohorts. **(A−C)** Data of the training cohort, **(D−F)** data of the testing cohort, **(G−I)** data of the entire cohort. **(A,D,G)** The ranked dot plot of risk score distribution. **(B,E,H)** The scatter plot of patients’ overall survival status. **(C,F,I)** The heatmap of expression profile of the six signature DDRGs.

**FIGURE 3:**
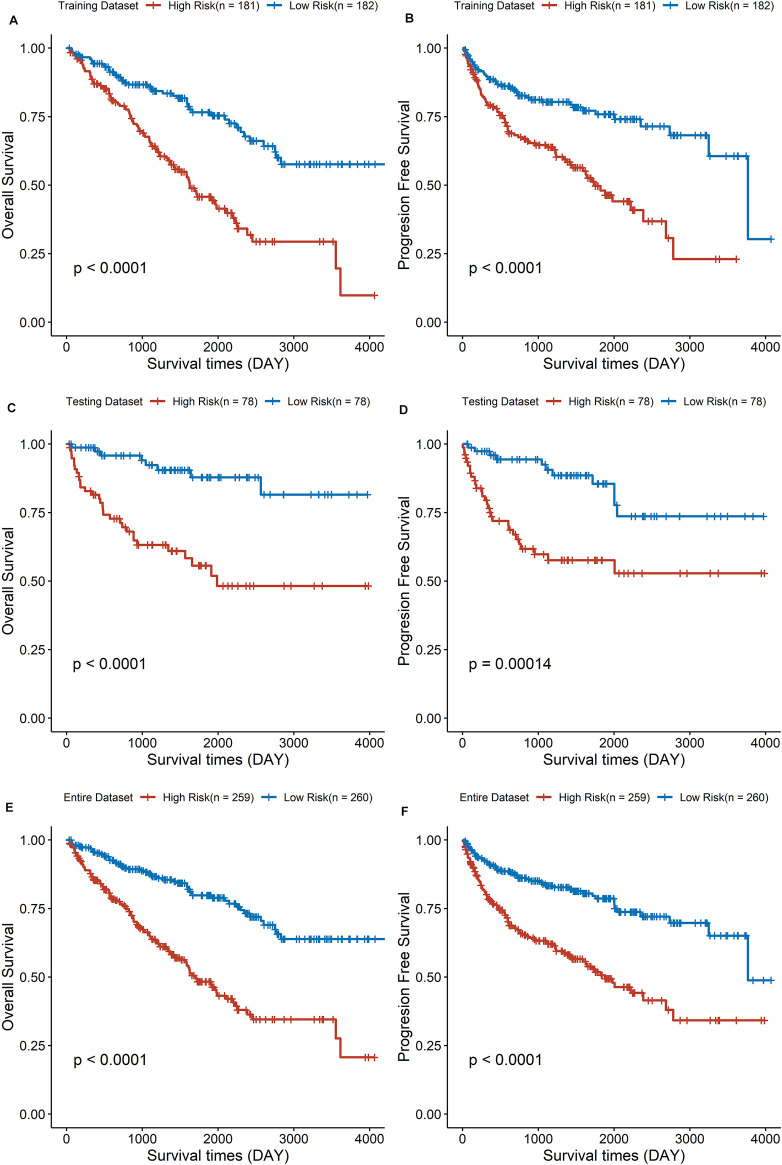
The survival analysis of overall survival and progression-free survival between low- and high-risk group in three cohorts. **(A,C,E)** Results of the overall survival analysis. **(B,D,F)** Results of the progression-free survival analysis.

Based on the median value of the risk score, the ccRCC cohort was divided into low- and high-risk groups. To testify the prognostic capability of this signature model, the training dataset, testing dataset, and entire dataset were compared using the uniform formula, respectively. Between low- and high-risk groups, the distribution of risk scores and the survival status of each ccRCC patient are depicted (Figures 2A, B,D, G,E, H). As shown in the heatmap of gene expression profile, MSH3 and RAD50 were downregulated, while NEIL3, RAD54L, EME1, and UNG were upregulated in the high-risk group (Figures 2C, F,I). Additionally, the differential expression of the six DDRGs showed that RAD54L, EME1, and NETL3 were upregulated in ccRCC tissue, while MSH3 and UNG were downregulated in ccRCC tissue compared with normal tissue (Supplementary Figure S1). In terms of OS and PFS, Kaplan–Meier survival analyses were performed on training, testing, and entire dataset. For all three datasets, both OS and PFS of the low-risk group were longer than that of the high-risk group (all *p* < 0.001) (Figure 3), which indicates that a high-risk score was associated with a poor clinical outcome.

### Enrichment of DNA Damage Repair Pathways in the High-Risk Group

**FIGURE 4:**
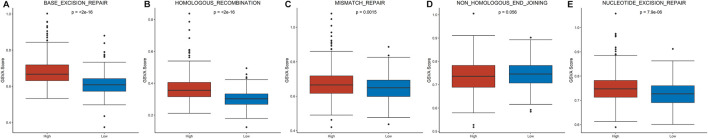
Gene set enrichment analysis of DNA repair pathways between the high- and low-risk group.

To explore patterns of DNA damage repair pathways potentially contributing to tumor progression, a total of five DNA damage repair pathways were compared between the high-risk and low-risk groups. Four pathways were significantly enriched in high-risk group (Figures 4A–C,E): base-excision-repair (*p* < 0.0001), homologous-recombination (*p* < 0.0001), mismatch-repair (*p* = 0.001) and nucleotide-excision-repair (*p* < 0.0001). However, no significant enrichment was detected in the pathway of non-homologous-end-joining (*p* = 0.056, Figure 4D).

### Functional Enrichment Analysis Between the High- and Low-Risk Groups

**FIGURE 5:**
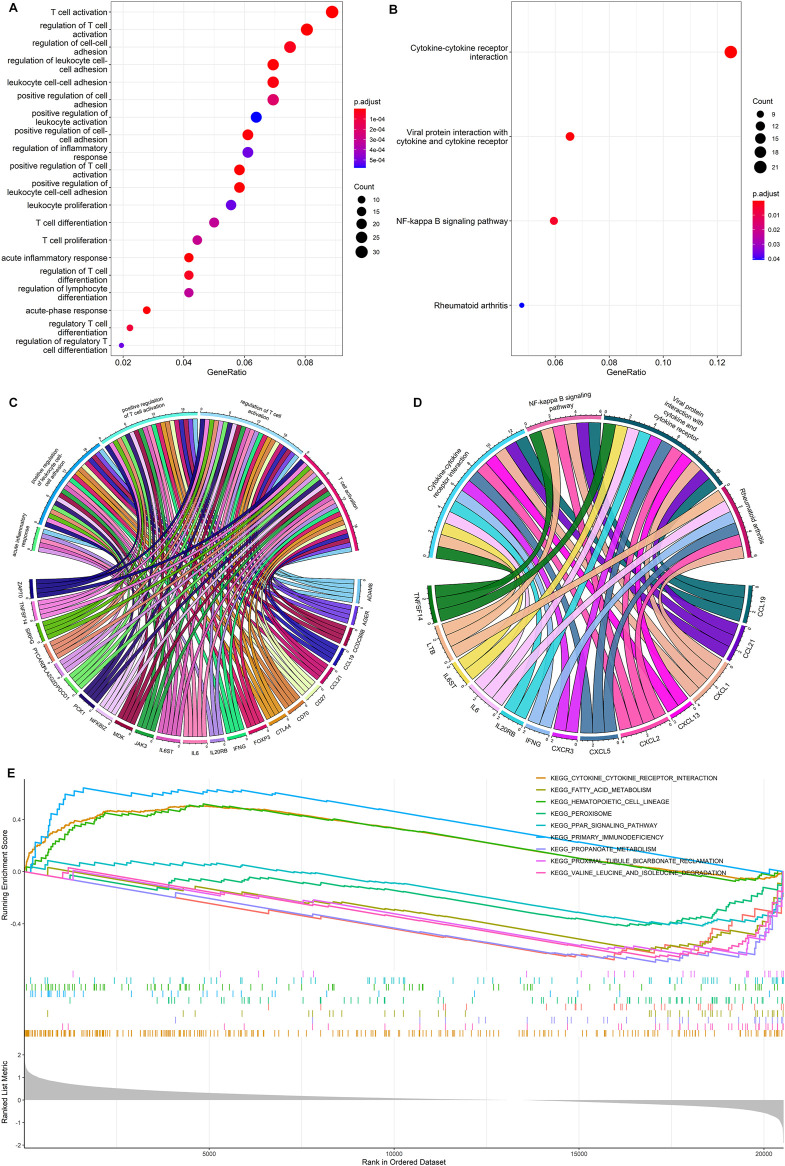
Functional enrichment analysis. **(A,C)** GO enrichment analysis for the DEGs between the high- and low-risk group. **(B,D)** KEGG enrichment analysis for the DEGs between the high- and low-risk group. **(E)** Gene set enrichment analysis for DEGs between the high- and low-risk groups.

We performed GO analysis to identify a total of 23 differentially expressed genes (DEGs) between high- and low-risk groups. The GO analysis mainly revealed the involvement of immune-related biological processes, including T-cell activation, regulation of T-cell activation, positive regulation of T-cell activation, positive regulation of leukocyte cell-cell adhesion, etc. (Figures 5A, C).

The KEGG pathway analysis showed that a total of 13 DEGs were mainly enriched in cytokine-cytokine receptor interactions, viral protein interaction with cytokine and cytokine receptor, NF-Kappa B signaling pathway, and rheumatoid arthritis (Figures 5B, D). The gene set enrichment analysis further demonstrated the primary immunodeficiency pathway was significantly enriched in the high-risk group (Figure 5E). Functional enrichment analysis indicates that DEGs between high- and low-risk groups are mainly associated with immune-related biological processes in ccRCC.

### Tumor Immune Microenvironment and Immunotherapy Response

**FIGURE 6:**
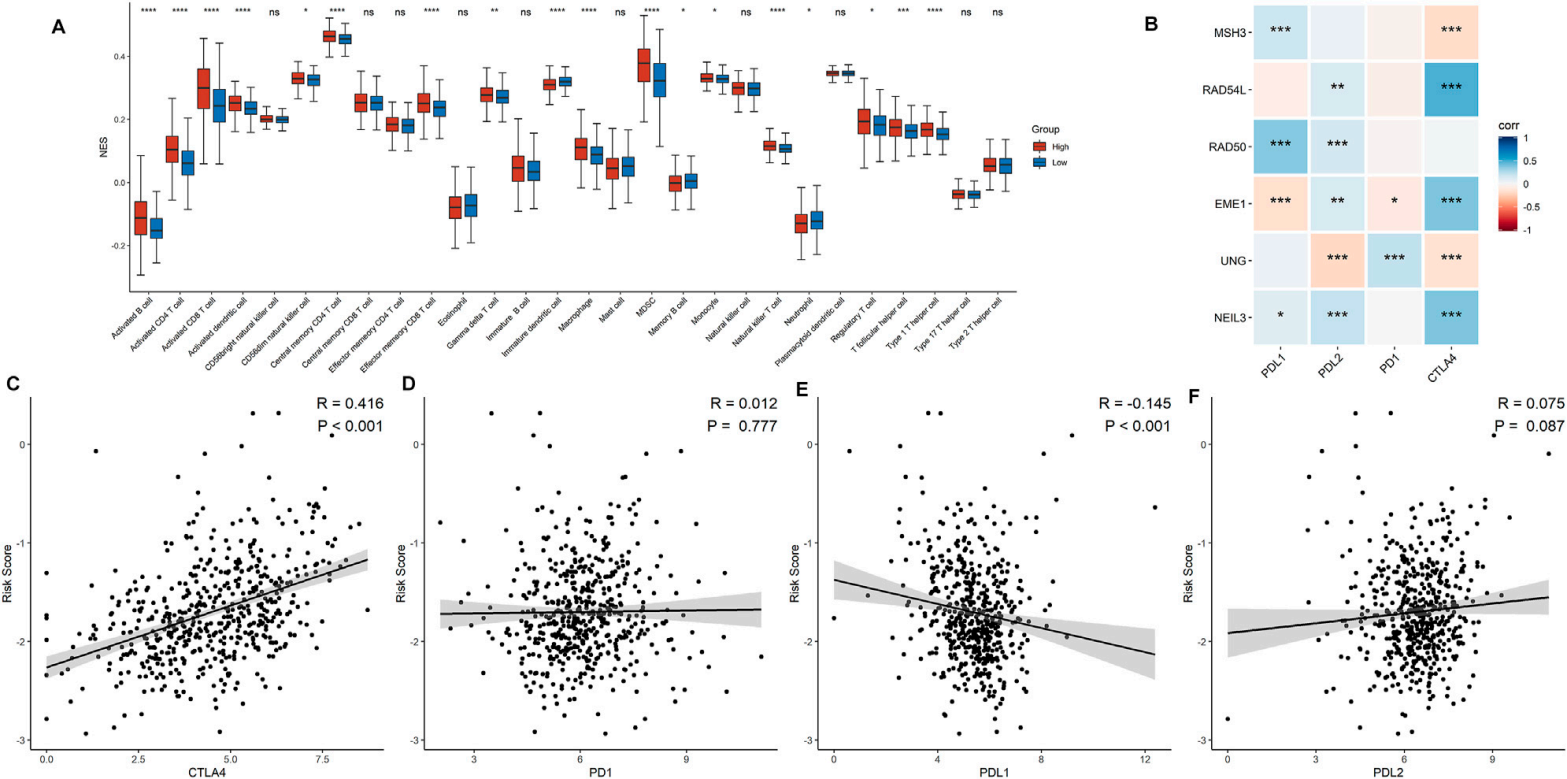
Association between the risk signature and immune-related characteristic. **(A)** The distribution of the immune cell infiltration levels between the low- and high-risk groups. **(B)** Correlation analysis for the six signature DDRGs with the different immune checkpoint markers. **(C−F)** Correlation analysis for the risk score and the immune checkpoint makers including CTLA4 **(C)**, PD-1 **(D)**, PD-L1 **(E)**, and PD-L2 **(F)**.

Correlations between DDRG signature and immune cell infiltration were further assessed based on 519 ccRCC samples. As shown in Figure 6A, prominent differences were shown in the infiltration of immune cells between low- and high-risk groups, which indicates that DDRG signature is significantly correlated with immune cell infiltration in ccRCC. For example, activated B cell, activated CD4^+^ cell, and activated CD8^+^ cell were significantly infiltrated in the high-risk group. However, the correlation between six DDRGs and the expression of immune markers demonstrated that PD-1 and PD-L1 were less expressed in DDRG signature (Figure 6B). Additionally, risk signature score was positively correlated with expression of CTLA4 (Figure 6C, *p* < 0.001), but negatively correlated with PD-L1 (Figure 6E, *p* < 0.001). No significant correlation was found in risk signature score with PD-1 (Figure 6D, *p* = 0.777) and PD-L2 (Figure 6F, *p* = 0.777).

**FIGURE 7:**
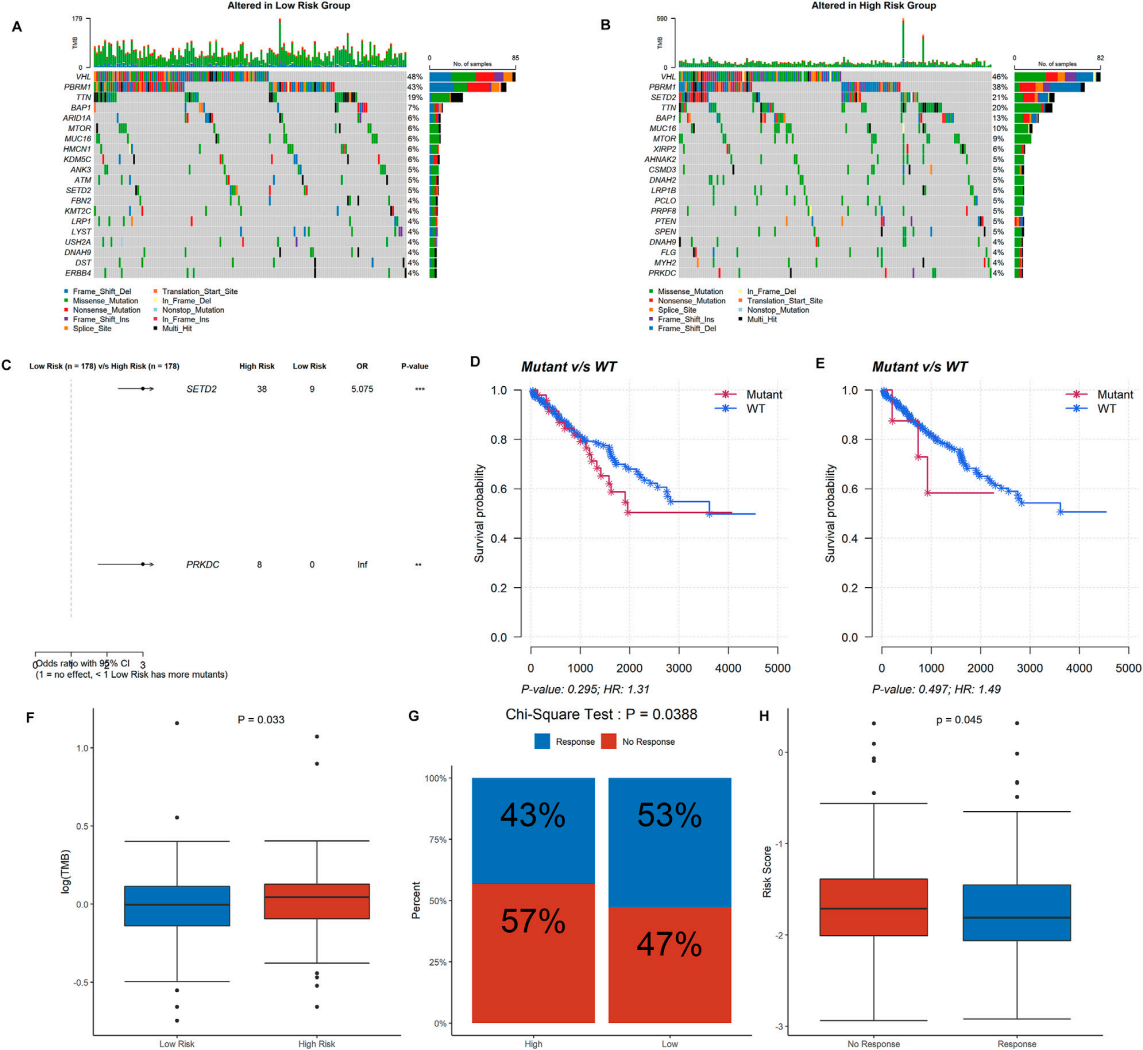
Information of mutation landscape analysis and immunotherapy response. **(A,B)** The top 20 driver genes with the highest alteration frequency in the low- and high-risk groups, respectively. **(C,D,E)** Significant mutation genes between two risk group and their survival analysis in ccRCC patients. **(F)** Tumor mutational burden between the low- and high-risk groups. **(G)** Comparison of immunotherapy response in the high- and low-risk groups. **(H)** Risk score between immunotherapy response group and no response group.

Mutation data from 356 ccRCC samples were then analyzed and summarized. The top 20 driver genes with the highest alteration frequency in the low- and high-risk groups are shown in Figures 7A, B. Additionally, we found that the mutation of SETD2 and PRKDC was more likely to occur in the high-risk group (Figure 7C), but the mutation of SETD2 and PRKDC was not associated with prognosis in ccRCC (Figures 7D, E). Tumor mutational burden (TMB) was higher in the high-risk group (Figure 7F).

ccRCC patients in the high-risk group were less likely to respond to immunotherapy than the low-risk group (43 *vs*. 53%, *p* = 0.039) (Figure 7G), as well as the response group owned a lower risk score (*p* = 0.045) (Figure 7H). The correlations between the DDRG signature and immunotherapeutic biomarkers indicate that the DDRG signature might serve as an indicator for immunotherapy response in ccRCC. Furthermore, we evaluated the role of each of the six DDRGs in immunotherapy response and expression of RAD50 and RAD54L were considered taking a positive role in immunotherapy response (*p* < 0.001 and *p* = 0.044, respectively, Supplementary Figure S2).

### Association Between the Risk Signature Score and the Clinicopathological Characteristics

**FIGURE 8:**
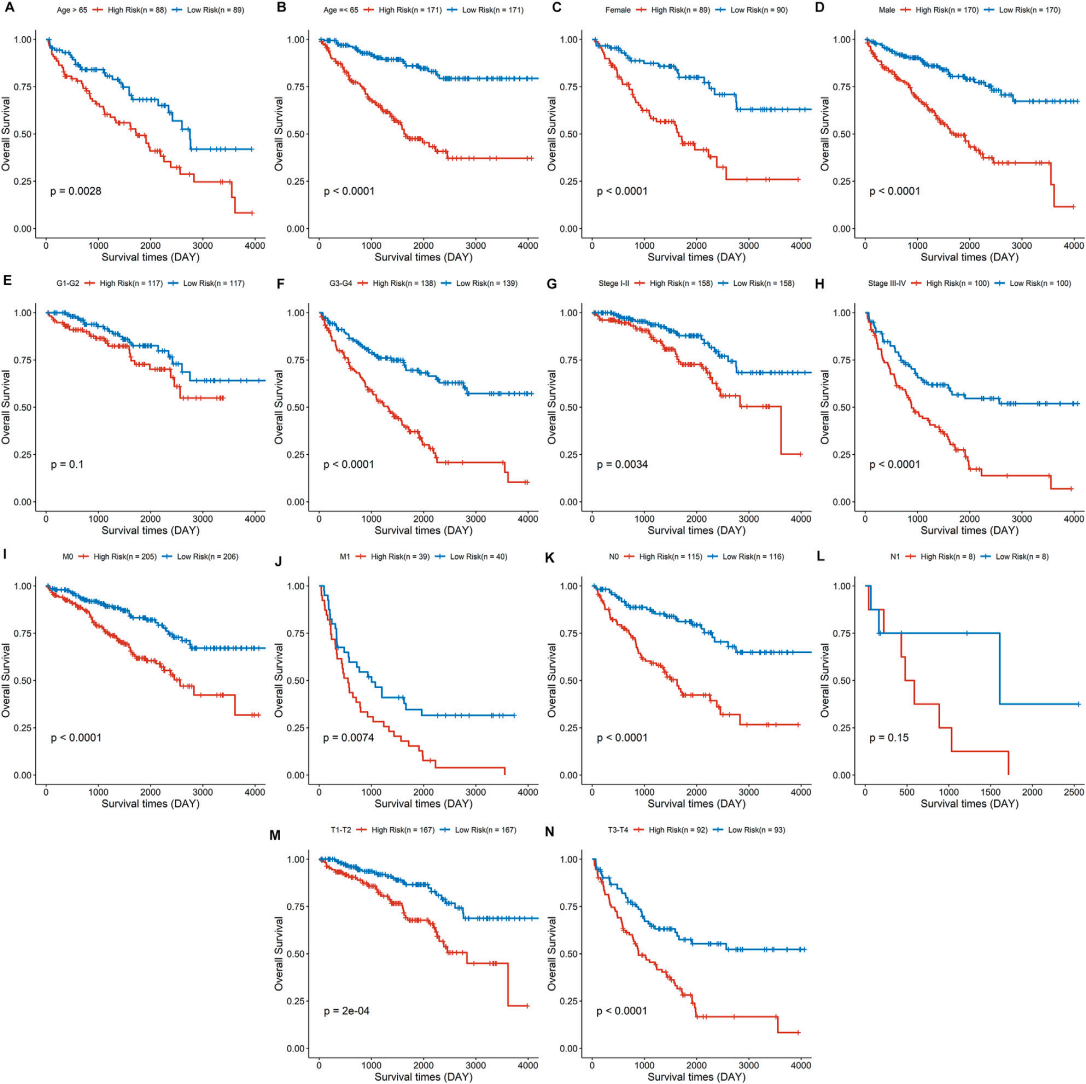
Survival analysis for the high- and low-risk group stratified by age **(A,B)**, sex **(C,D)**, pathological grade **(E,F)**, clinical stage **(G,H)**, distant metastasis **(I, J)**, lymph node invasion **(K,L)**, and tumor size **(M,N)**.

The discrepancies in OS stratified by clinicopathologic characteristics were analyzed between the low- and high-risk groups in the entire ccRCC dataset (Supplementary Table S1). Subgroups were classified by age, gender, pathological grade, clinical stage, distant metastasis, lymph node invasion, and tumor size. ccRCC patients with low-risk scores owned a longer overall survival time compared to those with a high-risk score in each subgroup (Figures 8A–D, F–K, M,N), except in the subgroups of pathological grade I-II and lymph node status I (Figures 8E, L). These findings indicate that the risk signature could be used to predict the overall survival of ccRCC patients without considering the impact of the clinical characteristic. Furthermore, a risk score based on DDRGs is closely related to the grade, stage, and TNM of clear cell renal cell carcinoma patients (all *p* < 0.001), indicating the viability of DDRGs in predicting ccRCC prognosis from another point (Supplementary Figure S3).

### Construction and Validation of the Prognostic Nomogram

**FIGURE 9:**
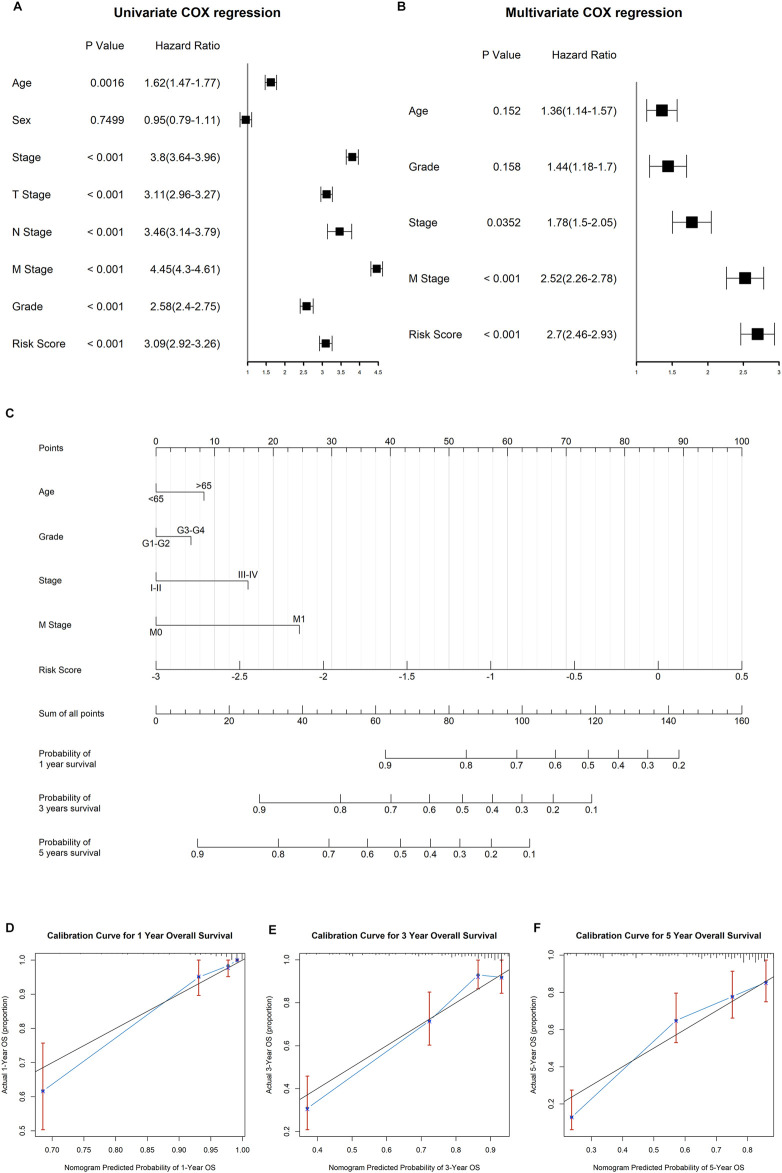
Construction of the nomogram to predict the overall survival for ccRCC patients. **(A)** Results of univariate cox regression analysis for OS in ccRCC cohort among risk signature score and clinical characteristics. **(B)** Results of stepwise multivariate cox regression analysis based on univariate cox regression. **(C)** Construction of a nomogram predicting the 1-, 3-, and 5-year OS for ccRCC patients. **(D−F)** Calibration curve for the nomogram predicting the 1-year **(A)**, 3-year **(B)**, and 5-year **(C)** OS in ccRCC.

Both univariate and multivariate Cox regression analyses were performed to evaluate whether the clinicopathological characteristics and risk score can serve as independent prognostic indicators for ccRCC patients. As shown in Figure 9A, age, clinical stage, TNM stage, pathological grade, and risk score were closely related to overall survival time of ccRCC. In the multivariate stepwise cox regression analysis (Figure 9B), we eventually found that age, pathological grade, clinical stage, distant metastasis, and risk score were independent prognostic factors, which were then used for the construction of the prognostic nomogram.

The established nomogram was fabricated to predict the 1-, 2-, and 3-year OS for ccRCC patients (Figure 9C). By comparison with clinical factors, the risk score showed great predictive ability in the nomogram. Furthermore, the calibration curves showed a consistent fitness between the predicted and observed values for 1-, 3-, and 5-year OS (Figures 9D–F).

## Discussion

Accumulating studies have shown that different renal cell carcinoma subtypes have distinct clinicopathological characteristics and prognoses. As the most common subtype of renal cell carcinoma, ccRCC possesses relatively poor clinical outcomes, especially when the advanced RCC were first diagnosed in the clinic (Shuch et al., 2015). When patients are presented with metastatic lesions or recurrence after complete surgical resection, chemotherapy and radiation therapy are the main options, but they are largely ineffective in renal cell carcinoma (Makhov et al., 2018). The lack of sensitivity to traditional systemic therapies in RCC calls for novel treatment strategies. More recently, immunotherapy or immune checkpoint inhibitors like PD-1 and PD-L1 have emerged as effective options for advanced ccRCC (Zarrabi et al., 2017). Based on the genomic signature, an increasing number of studies have been focused on forecasting the prognosis and immunotherapeutic response in patients with ccRCC. But clinical prognosis and personalized treatment based on DDRGs are rarely studied in ccRCC. In the present study, we constructed a DDRG risk signature and identified its role in clinical prognosis and immunotherapeutic response.

As we know, the stability of the DNA double-strand is significant to the viability of a normal cell, otherwise, loss of genetic material during mitosis or replication might occur. Mismatch repair, base excision repair, nucleotide excision repair, homologous recombination, and nonhomologous end-joining are the most common DNA damage repair methods, which are of great significance in maintaining genetic stability. Generally, cancer cells often harbor a poor capability of DNA repair and DNA-damage signaling, and cancers might also upregulate mutagenic repair pathways that drive oncogenesis (Jeggo and Löbrich, 2015). Consequently, a suboptimal DNA repair capability will eventually lead to replication stress and subsequent accumulation of DNA damage in cancer cells. Moreover, it has been reported that alteration in the DNA damage repair pathway was associated with increased recurrence in patients with ccRCC (Na et al., 2019).

In the present study, 105 DDRGs were analyzed in 519 ccRCC patients to explore the prognostic function of DDRGs. Univariate Cox regression revealed the prognostic value of 47 DDRGs, 6 of which were finally applied to construct the DDRG signature by LASSO-penalized Cox analysis. ccRCC patients were divided into high- and low-risk groups based on the risk score calculated by the DDRG signature. The high-risk group demonstrated a poor survival time compared to the low-risk group. Moreover, this risk signature showed perfect consistency in the testing dataset. This risk signature model involved six DDRGs, including MSH3, RAD54L, RAD50, EME1, UNG, and NEIL3, whose biological function in DNA damage repair has been previously reported. However, their role in ccRCC was rarely reported before. UNG (involved in uracil-DNA glycosylase) and NEIL3 (removes oxidative products of pyrimidines) participated in base excision repair. A mismatch and loop recognition, MSH3 is associated with mismatch excision repair. RAD50 (served as ATPase in complex with MRE11A and NBS1), RAD54L (served as accessory factors for recombination), and EME1 (as subunits of structure-specific DNA nuclease) are linked to homologous recombination.

It has been reported that the loss of NEIL3 in prostate cancer could inhibit cell apoptosis and cell cycle arrest under cisplatin treatment (Wang et al., 2021). UNG rs246079 G/A might contribute to a decreased risk of esophageal cancer and an increased risk of cervical carcinoma (Yin et al., 2014; Ye et al., 2018). Tumors with low Eme1 levels were more sensitive to cisplatin-based chemotherapy than tumors with high levels (Tomoda et al., 2009). Cancers with abnormal mismatch repair gene expression including MSH1 were associated with microsatellite instability-high (MSI-H) phenotype (Kaur et al., 2011). Low-level expression of RAD50 is associated with poor disease-free survival and OS in early-stage/low-grade rectal cancer patients (Ho et al., 2017). High RAD54L expression was linked to a shorter survival of patients with bladder cancer (Mun et al., 2020). However, the prognostic role of these six DDRGs is still unknown in ccRCC, as well as the risk signature we constructed.

Tumor cell escape from immune surveillance by upregulating the expression of PD-1 and PD-L1 in the tumor microenvironment, and immunotherapy activate immune activities by blocking the interaction of PD-1/PD-L1 (Tumeh et al., 2014). In this study, the DDRG signature was negatively correlated with an immune marker of PD-L1, and not correlated with expression of PD-1 and PD-L2, suggesting that patients with high-risk scores would have lower expression of these immune markers and might account for immunotherapy resistance. In the further computational prediction of immunotherapy response with the TIDE algorithm, we got a consistent result that low-risk signature scores tend to benefit from immunotherapy rather than a high-risk group. TMB as an effective biomarker to predict PD-L1 response has been revealed by accumulated studies (Chan et al., 2019). Surprisingly in our study, the TMB score was higher in the high-risk, which showed a negative correlation with immunotherapy response in ccRCC. These results indicate that the risk signature might serve as a predictor of immunotherapy response for ccRCC patient, but the role of TMB still required further investigations.

Currently, the pathological stage is the decisive factor in the prognosis of ccRCC. However, ccRCC patients at the same stage always have different clinical outcomes, reflecting the heterogeneity of ccRCC and calling for novel predictive and therapeutic biomarkers. The DDRG signature we constructed showed an excellent predictive ability as an independent prognostic indicator when compared to other clinicopathological characteristics. We also tried to establish a nomogram by combing DDRG signature scores and clinical characteristics, which showed the great capability to predict 1-, 3-, and 5-year OS for ccRCC patients.

In the present study, we selected several bioinformatic models and methods to testify to this DDRG signature. Based on current evidence, the explainability of this novel model was acceptable without validation from external data. However, limitations in the present study should also be noticed. First, external validation from local clinical datasets would be beneficial for further application. Additionally, the biological mechanism of DDRG signature has not been fully elucidated in the present study because of space limitations, the role of DDRGs in potential biological pathways, and tumor microenvironment should be testified in future studies.

## Conclusion

Our study identified a novel DDRG signature associated with prognostic outcomes and immunotherapeutic response in patients with ccRCC. Additionally, the nomogram comprising both DDRG signature and clinicopathological characteristics showed a good fitness to predict 1-, 3-, and 5-years overall survival for ccRCC patients.

## Data Availability

ccRCC Data generated and analyzed in the present study are available from the UCSC TCGA data portal (http://xena.ucsc.edu/public/). All data generated or analyzed during this study are included in this published article and its Supplementary Files. We have also uploaded the code and data to a public repository-GitHub. Both readers and reviewers could check up the computational process, as well as the original or merged data of this article following the link https://github.com/Linjie0120/DNA-damage-repair-genes-of-CCRCC.git.

## Abbreviations

ccRCC, Clear cell renal cell carcinoma; DDRGs, DNA damage repair genes; DEGs, differentially expressed genes; GO, Gene Ontology; KEGG, Kyoto Encyclopedia of Genes and Genomes; OS, overall survival; PFS, progress-free survival.
