# Supplementary material for: A DNA Damage Repair Gene Signature Associated With Immunotherapy Response and Clinical Prognosis in Clear Cell Renal Cell Carcinoma
Source: Front Genet. 2022 May 17;13:798846. doi: 10.3389/fgene.2022.798846 (PMC9152249; doi:10.3389/fgene.2022.798846)
Supplement: Supplementary file 1 [file Table1.DOCX]

**Supplementary table 1: Demographics of ccRCC dataset.**

| Variable | | N | High-risk group,  N = 259^1^ | Low-risk group,  N = 260^1^ | p-value^2^ |
| --- | --- | --- | --- | --- | --- |
| **Age** | | 519 |  |  | 0.37 |
| <65 | |  | 176 (68%) | 166 (64%) |  |
| >65 | |  | 83 (32%) | 94 (36%) |  |
| **Clinical Stage** | | 516 |  |  | <0.001 |
| I-II | |  | 133 (52%) | 183 (70%) |  |
| III-IV | |  | 123 (48%) | 77 (30%) |  |
| **T_ stage** | | 519 |  |  | <0.001 |
| T1-T2 | |  | 144 (56%) | 190 (73%) |  |
| T3-T4 |  | | 115 (44%) | 70 (27%) |  |
| **N_ stage** | 247 | |  |  | 0.019 |
| N0 |  | | 112 (90%) | 119 (98%) |  |
| N1 |  | | 13 (10%) | 3 (2.5%) |  |
| **M_ stage** | 490 | |  |  | <0.001 |
| M0 |  | | 184 (76%) | 227 (92%) |  |
| M1 |  | | 58 (24%) | 21 (8.5%) |  |
| **Sex** | 519 | |  |  | 0.046 |
| female |  | | 78 (30%) | 101 (39%) |  |
| male |  | | 181 (70%) | 159 (61%) |  |
| **Grade** | 511 | |  |  | <0.001 |
| G1-G2 |  | | 92 (36%) | 142 (56%) |  |
| G3-G4 |  | | 165 (64%) | 112 (44%) |  |
| ^1^n (%) | | | | | |
| ^2^Pearson's Chi-squared test; Fisher's exact test | | | | | |
