# Supplementary material for: A DNA Damage Repair Gene Signature Associated With Immunotherapy Response and Clinical Prognosis in Clear Cell Renal Cell Carcinoma
Source: Front Genet. 2022 May 17;13:798846. doi: 10.3389/fgene.2022.798846 (PMC9152249; doi:10.3389/fgene.2022.798846)
Supplement: Supplementary file 2 [file DataSheet1.docx]

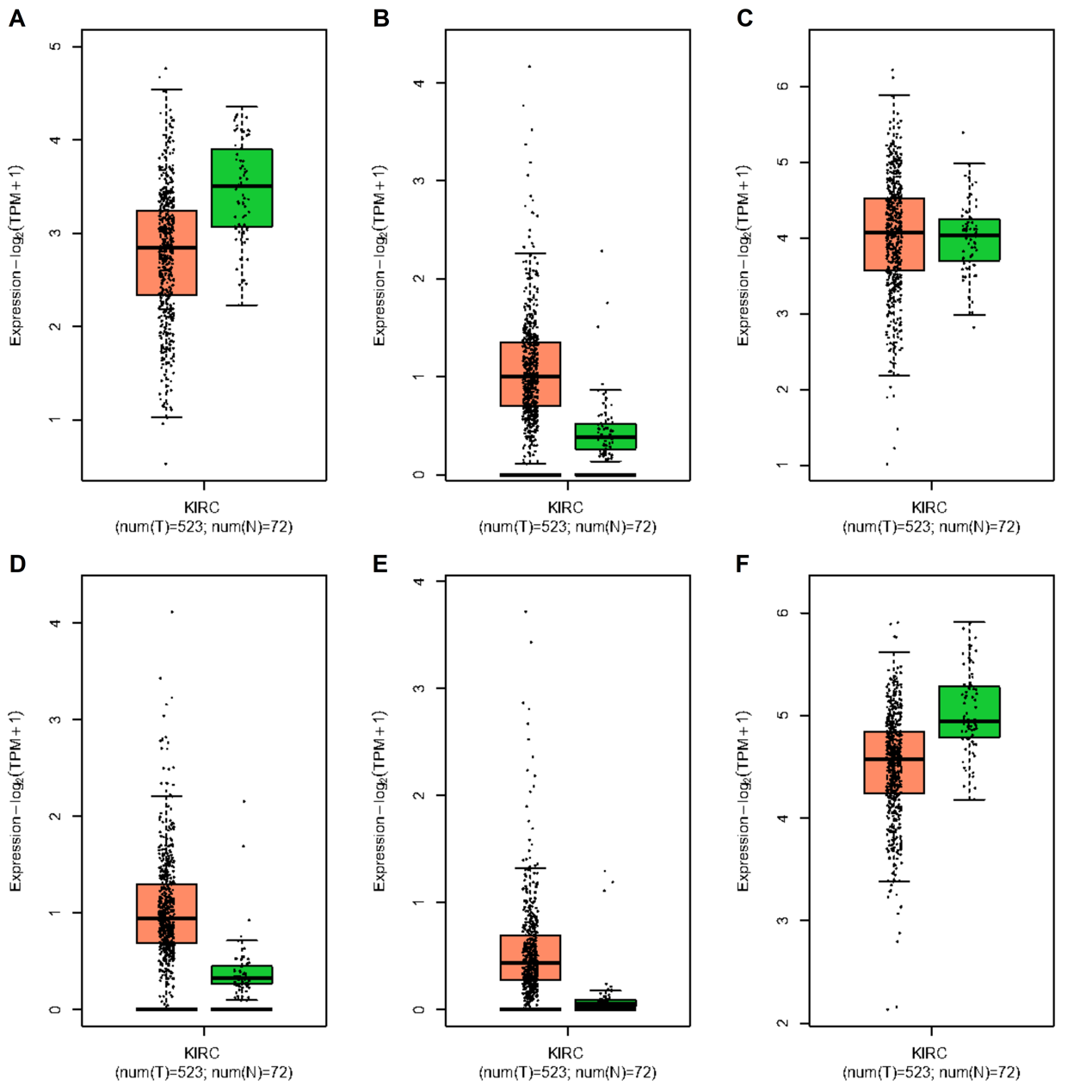


Figure S1. Differential expression of six DNA damage repaired genes between ccRCC and normal tissue. (A) Expression of MSH3 between tumor and normal tissue. (B) Expression of RAD54L between tumor and normal tissue. (C) Expression of RAD50 between tumor and normal tissue. (D) Expression of EME1 between tumor and normal tissue. (E) Expression of NETL3 between tumor and normal tissue. (F) Expression of UNG between tumor and normal tissue.


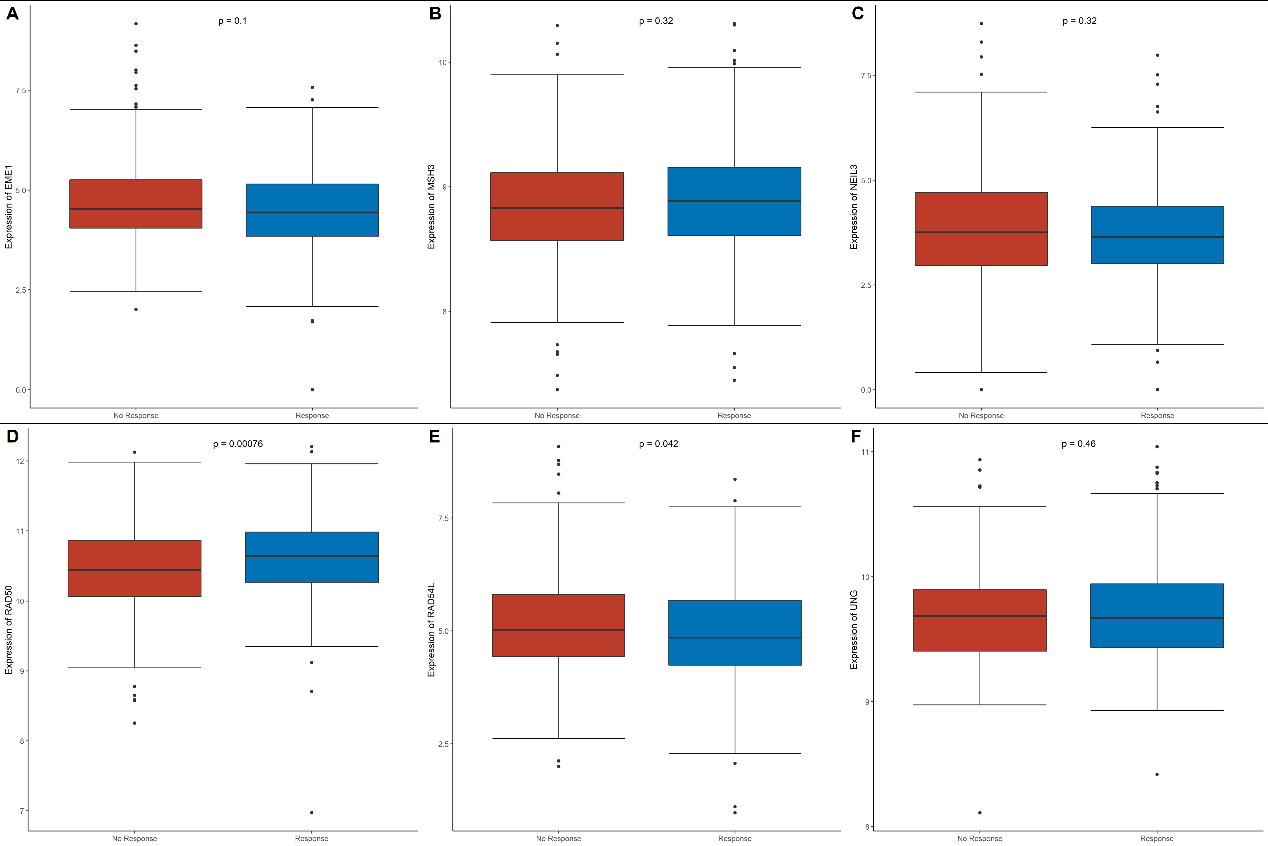


Figure S2. Relation between estimated immunotherapy response and six DNA damage repaired genes. (A) Expression of EME1. (B) Expression of MSH3. (C) Expression of NETL3. (D) Expression of RAD50. (E) Expression of RAD54L. (F) Expression of UNG.


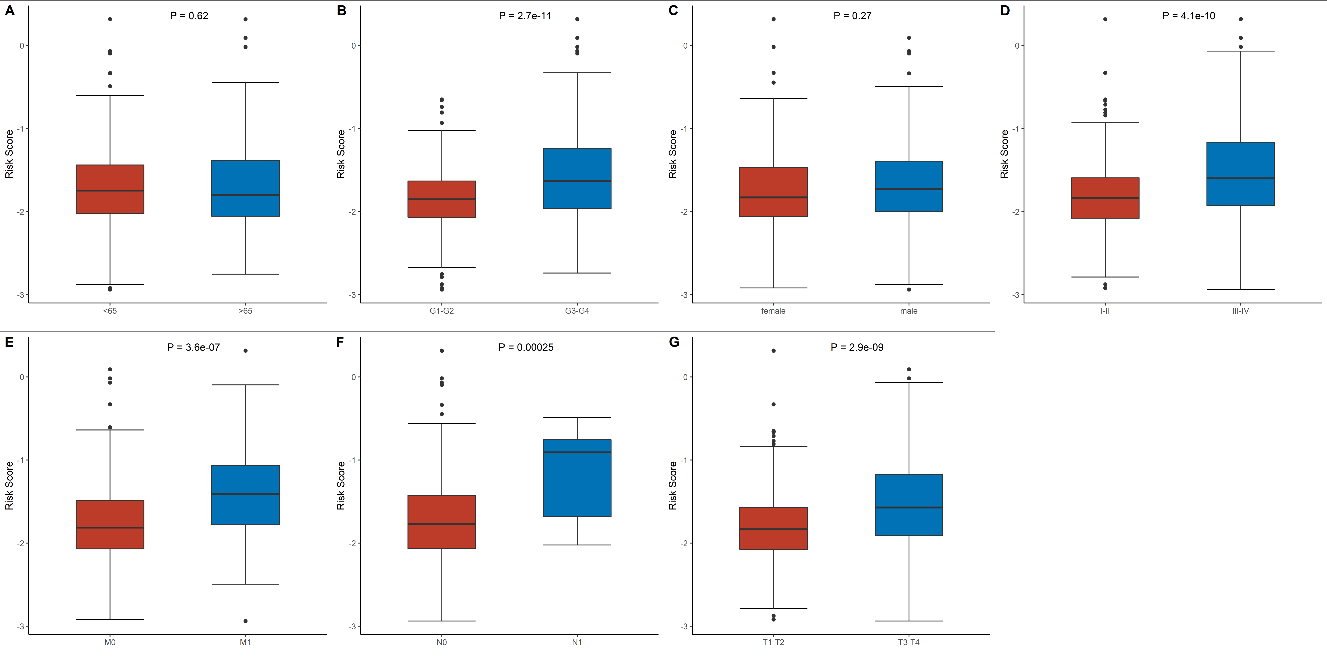


Figure S3. Relation between risk score and clinicopathological characteristics. (A) Relation with age. (B) Relation with grade. (C) Relation with sex. (D) Relation with stage. (E) Relation with M stage. (F) Relation with N stage. (G) Relation with T stage.
